# Discrete coalescent trees

**DOI:** 10.1007/s00285-021-01685-0

**Published:** 2021-11-05

**Authors:** Lena Collienne, Kieran Elmes, Mareike Fischer, David Bryant, Alex Gavryushkin

**Affiliations:** 1 Department of Computer Science, University of Otago, Dunedin, New Zealand; 2 Institute of Mathematics and Computer Science, University of Greifswald, Greifswald, Germany; 3 Department of Mathematics and Statistics, University of Otago, Dunedin, New Zealand; 4 School of Mathematics and Statistics, University of Canterbury, Christchurch, New Zealand

**Keywords:** Phylogenetics, Coalescent trees, Time trees, Metric geometry, Algorithms, Tree space, Tree distance, Ranked tree, 92B05, 05C85, 68Q25

## Abstract

In many phylogenetic applications, such as cancer and virus evolution, time trees, evolutionary histories where speciation events are timed, are inferred. Of particular interest are clock-like trees, where all leaves are sampled at the same time and have equal distance to the root. One popular approach to model clock-like trees is coalescent theory, which is used in various tree inference software packages. Methodologically, phylogenetic inference methods require a tree space over which the inference is performed, and the geometry of this space plays an important role in statistical and computational aspects of tree inference algorithms. It has recently been shown that coalescent tree spaces possess a unique geometry, different from that of classical phylogenetic tree spaces. Here we introduce and study a space of discrete coalescent trees. They assume that time is discrete, which is natural in many computational applications. This tree space is a generalisation of the previously studied ranked nearest neighbour interchange space, and is built upon tree-rearrangement operations. We generalise existing results about ranked trees, including an algorithm for computing distances in polynomial time, and in particular provide new results for both the space of discrete coalescent trees and the space of ranked trees. We establish several geometrical properties of these spaces and show how these properties impact various algorithms used in phylogenetic analyses. Our tree space is a discretisation of a previously introduced time tree space, called *t*-space, and hence our results can be used to approximate solutions to various open problems in *t*-space.

## Introduction

Most tree inference methods aim to reconstruct phylogenies with branch lengths representing times, so-called time trees. A popular assumption that is used for inferring clock-like time trees, where all leaves are sampled at the same time, is coalescent theory (Kingman 1982). The coalescent is widely employed for inferring relationships of a sample of genes (Hudson et al. 1990; Kuhner 2009), or for analysing population dynamics (Kuhner et al. 1998; Drummond et al. 2005). A recent striking application of coalescent theory is cancer phylogenetics (Posada 2020; Ohtsuki and Innan 2017), where accurate estimates of divergence times are essential for targeted treatment strategies. Under a coalescent model, evolution is considered backwards in time, and two lineages coalesce after a waiting time, which is to be estimated. The resulting trees are referred to as coalescent trees, which are ultrametric trees where internal nodes are assigned unique times. The coalescent is often used together with assuming a molecular clock to infer times. The strict molecular clock is the assumption that the mutation rate of a gene is approximately constant over time. This phenomenon is a prediction of neutral theory, and was first famously confirmed experimentally by Zuckerkandl and Pauling (1965). Afterwards, the molecular clock became a popular hypothesis, and various relaxations were developed (Kumar and Hedges 2016). Independent on how branch lengths are inferred, if samples are taken at the same time, the underlying phylogeny is clock-like when branch lengths are proportional to time, which makes the coalescent a reasonable assumption for inferring clock-like genealogies.

In phylogenetic trees, which display evolutionary relationships, internal nodes can be labelled with times, inferred assuming a molecular clock. Software packages for reconstructing those trees from data such as RNA, DNA, or protein sequences rely on a parameterisation of trees where internal nodes are equipped with times. Popular tree inference software used for this purpose are based on Maximum Likelihood (Kozlov et al. 2019; Nguyen et al. 2015; Tamura et al. 2011) or Bayesian methods (Bouckaert et al. 2014; Suchard et al. 2018; Ronquist and Huelsenbeck 2003). They rely on tree search algorithms, where in every step a new tree is proposed and accepted if the proposed tree fulfils certain requirements. For tree proposals under the molecular clock assumption a parameterisation of trees taking the times of internal nodes into account is required. Furthermore, a similarity measure for these trees is necessary, to propose trees that are are measurably similar to a given tree in tree search algorithms.

Tree spaces that take branch lengths of trees into account have already been explored in the literature. One important example, the BHV-space (Billera et al. 2001), consists of orthants, representing tree topologies. Trees are parametrised by their branch lengths. This parameterisation is however not suitable for time trees because changing the times of an evolutionary event in the tree implies that all preceding events change their times as well. Hence two trees can be close to each other in this space even though the timing of many internal nodes is different in the two trees. Furthermore, subspaces of the BHV-space associated with different ranked topologies have different volumes, which makes it hard to introduce a probability distribution over such space without biasing towards certain ranked topologies. A detailed discussion of this topic can be found in Gavryushkin and Drummond (2016), where more suitable spaces for time trees, the *t*-space and $\tau$-space, are introduced and studied.

The *t*-space is a simplicial complex, where each simplex corresponds to a ranked tree topology and trees are parametrised by the actual time assigned to internal nodes. The $\tau$-space is made out of orthants that correspond to ranked tree topologies and time differences between consecutive nodes are used to parameterise trees. It has been observed, that in the $\tau$-space, like the BHV-space, shortest paths between trees often contain the star tree (Gavryushkin and Drummond 2016). This can be problematic in applications, for example when a pair of trees share some evolutionary information in form of a subtree, but this information is not preserved on a shortest path between them. For summary trees based on distance measures, this might result in losing such information shared between trees in the summary tree. Because of this, BHV- and $\tau$-space are not suitable for most applications, even though shortest paths can be computed efficiently. Although the *t*-space is free from these properties, no algorithm for computing distances or shortest paths between trees in this space is known yet, so applications are limited.

Enabling statistical analysis over the space of phylogenetic trees was an important motivation for Billera et al. (2001) to introduce the BHV-space and study its geometric properties. Tree space geometry has also played an important role in studies of rogue taxa in a tree (Cueto and Matsen 2011) and also summary trees (Miller et al. 2015). Here, driven by the same motivation, we propose to study coalescent trees. The geometric properties we study include a cluster property, which a tree space has if all shortest paths between two trees sharing a cluster preserve this cluster. A tree space with this property is desirable for constructing summary trees, as summaries for a set of trees sharing a cluster should also contain that cluster, a property that BHV-space and $\tau$-space do not have, as summary trees often end up being start trees. Clusters have also shown to play an important role in the development of algorithms for computing distances between trees (Bordewich and Semple 2005), as well as in constructing phylogenetic networks from trees (Baroni et al. 2006).

In this paper we introduce the space ${\mathrm {DCT}}_m$ of discrete coalescent trees, where internal nodes are assigned unique discrete times, and the time of the root is bounded from above by the integer *m*. This tree space is a discrete version of the *t*-space. ${\mathrm {DCT}}_m$ is also a generalisation of the ranked nearest neighbour interchange (${\mathrm {RNNI}}$) space (Collienne and Gavryushkin 2021). Here we show that the space ${\mathrm {DCT}}_m$ as well as ${\mathrm {RNNI}}$ have the desired properties mentioned above, including efficiently computable shortest paths that preserve biological information shared between trees. After introducing notations used throughout this paper (Sect. 2), we discuss how the algorithm ${\textsc {FindPath}}$ (Collienne and Gavryushkin 2021), originally designed for trees in ${\mathrm {RNNI}}$, can be generalised for the discrete coalescent tree space to compute shortest paths in polynomial time (Sect. 3). We then analyse some geometrical properties of both tree spaces ${\mathrm {DCT}}_m$ and ${\mathrm {RNNI}}$ (Sect. 4). First, we discuss the cluster property in Sect. 4.1 and then consider a subset of trees (caterpillar trees) for which we are able to compute ${\mathrm {RNNI}}$ distances more efficiently than with ${\textsc {FindPath}}$ (Sect. 4.2). Following that, we establish the diameter of ${\mathrm {DCT}}_m$ and ${\mathrm {RNNI}}$ and briefly discuss the radius for each space. We finish this paper with a conclusion and propose directions for further research (Sect. 5).

## Technical introduction

A *rooted binary phylogenetic tree* is a binary tree with *n* leaves uniquely labelled by elements of a set $\{a_1, \ldots , a_n\}$. The main object of study in this paper are *discrete coalescent trees*, binary rooted phylogenetic trees with a positive integer-valued *time* assigned to each node. More specifically, all *n* leaves $a_1, \ldots , a_n$ are assigned time 0, and every internal node is assigned a unique time less or equal to an integer *m*, such that it always has time greater than its children. We hence consider time going backwards from the leaves towards the root. Note that this implies $m \ge n-1$. We denote the time of an internal node *v* by ${\mathrm {time}}(v)$. The *length* of an edge (*u*, *v*)in a discrete coalescent tree, where *u* is parent of *v*, is the time difference of the nodes bounding the edge: ${\mathrm {time}}(u) - {\mathrm {time}}(v)$. If not stated otherwise, we refer to discrete coalescent trees simply as *trees*. We furthermore call two trees (not necessarily binary) *identical* if there is a graph isomorphism between them preserving leaf labels and times.

**Fig. 1:**
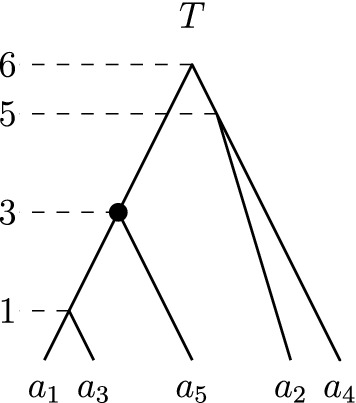
Discrete coalescent tree with $n = 5$ leaves and root height $m = 6$. The highlighted node with time three can be referred to as $(a_5)_T$ or $(\{a_1,a_5\})_T$, and the cluster induced by this node is $(T)_3$

As a special case of discrete coalescent trees we consider *ranked trees* with root time $n-1$. In these trees internal nodes have distinct times ranging from 1 to $n-1$. This definition of ranked trees coincides with the one of Collienne and Gavryushkin (2021). In the case of ranked trees we say the *rank* of a node *v* to mean its time (${\mathrm {rank}}(v) = {\mathrm {time}}(v)$) to be consistent with notations used in Collienne and Gavryushkin (2021). There are $\frac{(n-1)!n!}{2^{n-1}}$ ranked trees (Semple and Steel 2003). Every ranked tree gives ${m \atopwithdelims ()n-1}$ discrete coalescent trees, as every $(n-1)$-element subset of $\{1, \ldots , m\}$ can be the set of times assigned to the internal nodes of a ranked tree. Hence there are, contrary to the claim in Gavryushkin et al. (2018), $\frac{(n-1)!n!}{2^{n-1}} {m \atopwithdelims ()n-1}$ discrete coalescent trees.

Every internal node *v* of a tree *T* can be referred to by the set *C* of leaves that are descending from this node. We call such a set *C* *cluster* and say that the cluster *C* is *induced* by *v*. A list of clusters $[C_1, \ldots , C_{n-1}]$ determines at most one ranked tree (Collienne and Gavryushkin 2021), where cluster $C_i$ is induced by the internal node with rank *i* for $i \in \{1, \ldots , n-1\}$. For discrete coalescent trees however, times of nodes also need to be provided to uniquely identify a tree. For a subset $S \subseteq \{a_1, \ldots , a_n\}$ we call the internal node of a tree *T* with lowest time among those ancestral to all elements of *S* the *most recent common ancestor* of *S* and denote it by $(S)_T$. We furthermore denote the parent of a leaf $a_i$ in *T* by $(a_i)_T$, and the cluster induced by the node with time *i* in *T* by $(T)_i$. The node highlighted in Fig. 1 for example can be referred to as $(a_5)_T$, the parent of $a_5$, or $(\{a_1, a_5\})_T$, the most recent common ancestor of $\{a_1, a_5\}$, or of $(T)_3$, the cluster induced by the node with time three in *T*. Note that we will simply write ${\mathrm {rank}}(a_i)_T$ or ${\mathrm {time}}(a_i)_T$ to mean ${\mathrm {rank}}((a_i)_T)$ or ${\mathrm {time}}((a_i)_T)$, respectively. Although differing from traditional notation, this notation is intuitive, shortens nested formulas, and is consistent with notations used in Collienne and Gavryushkin (2021). A type of trees that will be of importance throughout the whole paper are *caterpillar trees*, which are trees where every internal node has at least one child that is a leaf. This implies that caterpillar trees have only one internal node that has two children that are leaves. In any tree, we call such a subtree with two leaves sharing a parent a *cherry*.

**Fig. 2:**
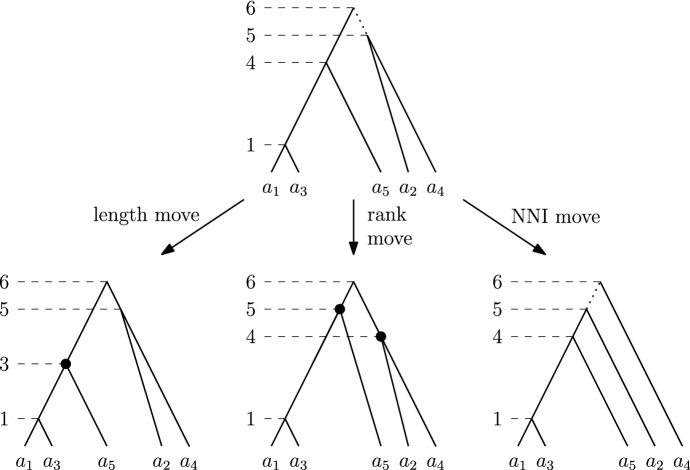
The three possible types of moves on a discrete coalescent tree: a length move changing the time of the highlighted node on the left, a rank move swapping the ranks of the highlighted nodes in the middle and an ${\mathrm {NNI}}$ move on the dotted edge on the right

We are now ready to introduce the central object of study of this paper, the graph (or space) of discrete coalescent trees. This graph is called ${\mathrm {DCT}}_m$ for a fixed positive integer *m*. The vertex set of ${\mathrm {DCT}}_m$ is the set of trees on *n* leaves with root time less or equal to *m*. Note that we assume the number of leaves *n* of the trees in the graph ${\mathrm {DCT}}_m$ to be fixed throughout this paper. Trees *T* and *R* are connected by an edge (*T* and *R* are *neighbours*) in this graph if performing one of the following (reversible) operations on *T* results in *R* (Fig. 2):

1. An ${\mathrm {NNI}}$ *move* connects trees *T* and *R* if there is an edge *e* in *T* and an edge *f* in *R*, both of length one, such that contracting *e* and *f* results in identical trees.
2. A *rank move* on *T* exchanges the times of two internal nodes with time difference one.
3. A *length move* on *T* changes the time of an internal node by one.

A length move can only change the time of a node to become *t* if there is no node with time *t* already. Furthermore, the time of the root of a tree in ${\mathrm {DCT}}_m$ cannot be changed by a length move to become greater than *m* in ${\mathrm {DCT}}_m$. Note that our definition of ${\mathrm {DCT}}_m$ differs from the definition of the space on discrete time-trees of Gavryushkin et al. (2018). In contrast to their definition, length moves in ${\mathrm {DCT}}_m$ do not change the height of a tree, unless it is performed on the root.

The definition of ${\mathrm {DCT}}_m$ leads to a natural definition of the *distance* between two trees *T* and *R* in this graph as the length of a shortest paths between these trees, denoted by $d_{{\mathrm {DCT}}}(T,R)$. We write a path in ${\mathrm {DCT}}_m$ as a list of trees $[T_0, T_1, \ldots , T_k]$, such that $T_i$ and $T_{i+1}$ are neighbours in ${\mathrm {DCT}}_m$, and denote the length *k* of such a path *p* by |*p*|. We also consider the ranked nearest neighbour interchange (${\mathrm {RNNI}}$) graph of Collienne and Gavryushkin (2021), which is the graph ${\mathrm {DCT}}_m$ for $m=n-1$, and hence a graph of ranked trees. In this graph length moves are not possible, so we use the notion ${\mathrm {RNNI}}$ *move* to mean either a rank move or an ${\mathrm {NNI}}$ move in order to distinguish these moves from length moves.

## Computing shortest paths in ${\mathrm {DCT}}_m$

Shortest paths, and therefore distances, between trees in ${\mathrm {RNNI}}$ can be computed with the algorithm ${\textsc {FindPath}}$, which was introduced by Collienne and Gavryushkin (2021) and has running time quadratic in the number of leaves *n*. As ${\mathrm {RNNI}}$ is a special case of ${\mathrm {DCT}}_m$ for $m = n-1$, the question arises whether a modification of this algorithm can also be used to compute shortest paths in ${\mathrm {DCT}}_m$. In this section we first describe ${\textsc {FindPath}}$ in ${\mathrm {RNNI}}$ before we present a generalisation of this algorithm for ${\mathrm {DCT}}_m$. For this generalisation we introduce a way to convert trees in ${\mathrm {DCT}}_m$ on *n* leaves into ranked trees on $m+2$ leaves, such that the ${\mathrm {RNNI}}$ distance between those ranked trees equals their distance in ${\mathrm {DCT}}_m$ (Theorem 1). We end this section by providing the algorithm ${\textsc {FindPath}}^+$, a version of ${\textsc {FindPath}}$ to work in the ${\mathrm {DCT}}_m$ without needing to transform discrete coalescent trees into ranked trees.

The algorithm ${\textsc {FindPath}}$ (Algorithm 1) constructs a path between two ranked trees *T* and *R* in ${\mathrm {RNNI}}$, which we denote by ${\mathrm {FP}}(T,R)$. This path is constructed iteratively from the initial path *p* only consisting of the tree $T_1 = T$ by adding new trees to the end of the path in every step. In each iteration $i = 1, \ldots , n-2$ (line 2 in Algorithm 1), the cluster $C_i$ induced by node of rank *i* in *R* is considered, and the rank of its most recent common ancestor in $T_1$ is decreased by one. This is either done by an ${\mathrm {NNI}}$ move (line 5), or a rank move (line 7), depending on the length of the edge below the most recent common ancestor (line 4). It has been shown that this move decreasing the rank of the most recent common ancestor is unique in every step (Collienne and Gavryushkin 2021, Proposition 1). After every such move, $T_1$ is updated to be the tree resulting from this move and *p* is extended by $T_1$. The tree at the end of iteration *i* contains the clusters $C_1, \ldots , C_i$, induced by nodes with rank up to (and including) *i* in *R*, and hence *p* is a path connecting *T* and *R*. The proofs that ${\textsc {FindPath}}$ is a deterministic algorithm and computes shortest paths in ${\mathrm {RNNI}}$ in ${\mathcal {O}}(n^2)$ can be found in Collienne and Gavryushkin (2021).
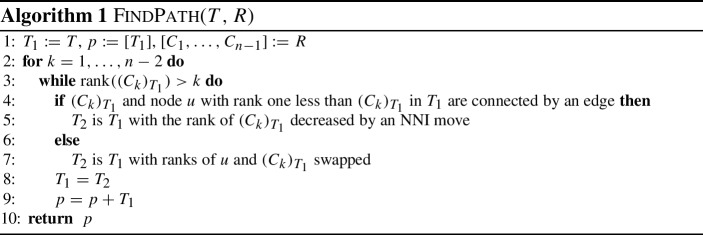


In Lemma 1 we show an important property of paths computed by ${\textsc {FindPath}}$. For two trees *T* and *R* and a cluster *C* that is present in both *T* and *R*, we say that a path *p* from *T* to *R* *preserves* *C* if every tree on *p* contains *C*.

### Lemma 1

For two ranked trees *T* and *R* in ${\mathrm {RNNI}}$ sharing a cluster *C*, the path ${\mathrm {FP}}(T,R)$ preserves this cluster *C*.

### Proof

**Fig. 3:**
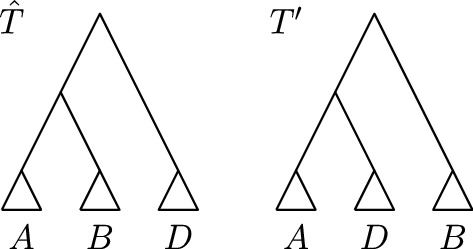
Ranked trees ${\hat{T}}$ and $T'$ as described in the proof of Lemma 1. The cluster $C = A \cup B$ is present in ${\hat{T}}$, but not in $T'$

We assume that *T* and *R* are ranked trees that share a cluster *C* and prove the lemma by contradiction. We therefore assume that there is a ranked tree on ${\mathrm {FP}}(T,R)$ that does not contain *C*. Let $T'$ be the first ranked tree on ${\mathrm {FP}}(T,R)$ that does not have *C* as a cluster. Since rank moves only change the ranks of internal nodes, i.e. the order of clusters in the cluster representation of a ranked tree, only ${\mathrm {NNI}}$ moves can actually change clusters. There must hence be an ${\mathrm {NNI}}$ move on ${\mathrm {FP}}(T,R)$ connecting a tree ${\hat{T}}$ that contains the cluster *C* with $T'$. Let *A* and *B* be the clusters induced by the children of the node inducing *C* in ${\hat{T}}$, meaning that $A \cup B = C$. Let furthermore *D* be the cluster induced by the node in ${\hat{T}}$ that shares its parent with the node inducing *C*. We can assume without loss of generality that the cluster *C* in ${\hat{T}}$ is replaced by the cluster $A \cup D$, as depicted in Fig. 3, as we can otherwise swap the notations for *A* and *B*.

From the move between ${\hat{T}}$ and $T'$ we can follow that the cluster in *R* whose most recent common ancestor is moved down by this move is a subset of $A \cup D$. In particular, this cluster contains elements in both *A* and *D*, but none in *B*. Since clusters in a ranked tree are always the union of two already existing clusters, a cluster and one leaf, or two leaves, a cluster in *R* that contains all elements of *A* and *B* must also contain elements of *D*. This however means that the cluster $C = A \cup B$ is not present in *R*, which contradicts our assumptions. We can therefore conclude that there cannot be a ranked tree $T'$ on ${\mathrm {FP}}(T,R)$ that does not contain *C*. $\square$

Note that Lemma 1 does not imply that every shortest path in ${\mathrm {RNNI}}$ preserves clusters. In Theorem 2 we will however see that this more general statement is actually true in ${\mathrm {RNNI}}$.

**Fig. 4:**
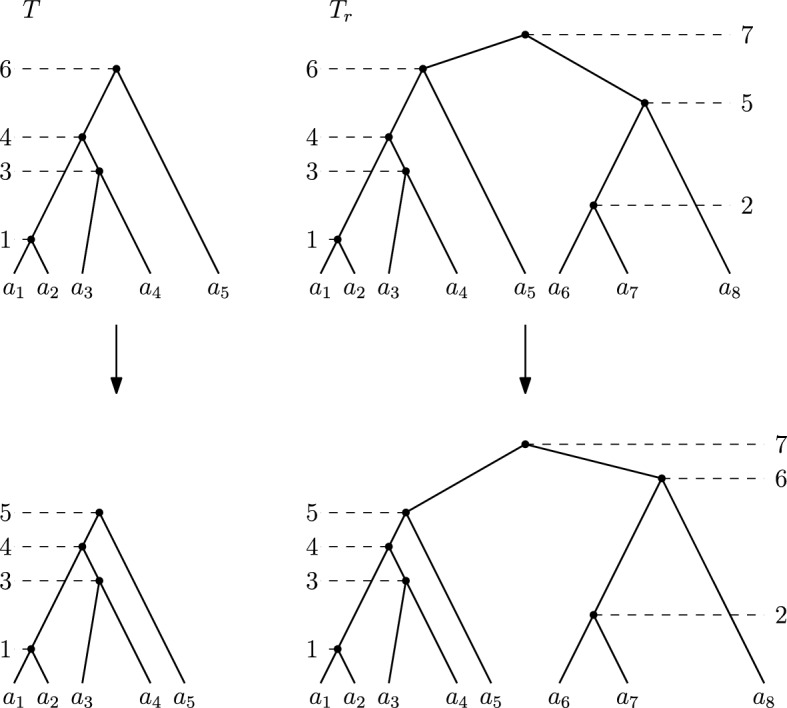
Extending a tree *T* on *n* leaves in ${\mathrm {DCT}}_6$ (top left) to a ranked tree with $m+2=8$ leaves (top right) by adding a caterpillar subtree with three leaves. The trees on the bottom result from *T* and $T_r$ by performing a length move (left) or rank move (right), respectively

Before we show how ${\textsc {FindPath}}$ works for discrete coalescent trees, we introduce a way to extend a tree *T* in ${\mathrm {DCT}}_m$ on *n* leaves into a ranked tree in ${\mathrm {RNNI}}$ with $m+2$ leaves (Algorithm 2). First add a new root with time $m + 1$ that becomes the parent of the root of *T*. The other child of this new root becomes the root of a caterpillar tree ${T_r}^c$ on leaf set $\{a_{n+1}, a_{n+2}, \ldots , a_{m+2}\}$, such that ${\mathrm {time}}(a_{n+1})_{{T_r}^c} = {\mathrm {time}}(a_{n+2})_{{T_r}^c}< {\mathrm {time}}(a_{n+3})_{{T_r}^c}< \ldots< {\mathrm {time}}(a_{m+2})_{{T_r}^c} < m+1$. An example of this extension of a tree *T* to a ranked tree $T_r$ is depicted in Fig. 4.

Throughout this paper we call this extension of a discrete coalescent tree *T* to a ranked tree the *extended ranked version* of *T* and denote it by $T_r$. Moreover, we denote the subtree of $T_r$ that is identical to *T* by $T_r^d$ (*d* for discrete coalescent tree) and the caterpillar subtree on leaf set $\{a_{n+1}, \ldots , a_{m+2}\}$ by $T_r^c$.
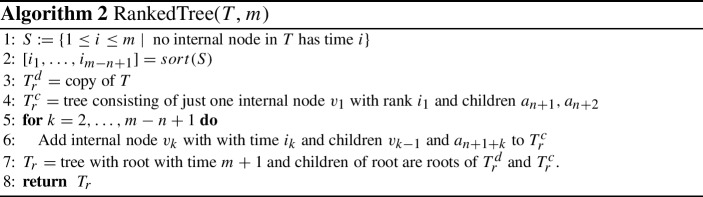

After extending both trees *T* and *R* in ${\mathrm {DCT}}_m$ to ranked trees $T_r$ and $R_r$ on $m+2$ leaves, respectively, we can compute shortest paths between $T_r$ and $R_r$ in ${\mathrm {RNNI}}$, using ${\textsc {FindPath}}$. We denote the path that ${\textsc {FindPath}}$ computes for two ranked trees $T_r$ and $R_r$ by ${\mathrm {FP}}(T_r,R_r)$. In Theorem 1 we show that the length of ${\mathrm {FP}}(T_r,R_r)$ in ${\mathrm {RNNI}}$ is equal to the distance of *T* and *R* in ${\mathrm {DCT}}_m$. Note that for any given pair of trees *T* and *R*, we always assume *m* to be greater or equal to the maximum root time of these trees and consider a shortest path between them in ${\mathrm {DCT}}_m$.

### Theorem 1

Let *T* and *R* be discrete coalescent trees and $T_r$ and $R_r$ their extended ranked versions. The distance $d_{\mathrm {DCT}}(T,R)$ between *T* and *R* in ${\mathrm {DCT}}_m$ equals the distance $d_{\mathrm {RNNI}}(T_r,R_r)$ between their extended ranked versions in ${\mathrm {RNNI}}$, where *m* is greater or equal to the maximum root height of *T* and *R*.

### Proof

We prove this theorem by showing that the following two inequalities hold for two discrete coalescent trees *T* and *R* and a shortest path *p* in ${\mathrm {DCT}}_m$ between those trees: $|p| \ge |{\mathrm {FP}}(T_r,R_r)|$ and $|p| \le |{\mathrm {FP}}(T_r,R_r)|$. Since we know that ${\textsc {FindPath}}$ computes the ${\mathrm {RNNI}}$ distance for ranked trees, it then follows $d_{{\mathrm {DCT}}}(T,R) = |p| = |{\mathrm {FP}}(T_r,R_r)| = d_{{\mathrm {RNNI}}}(T_R,R_r)$.

1. $$|p| \ge |{\mathrm {FP}}(T_r,R_r)|$$
  We use *p* to construct a path $p'$ between the extended ranked versions $T_r$ and $R_r$ of *T* and *R* with length $|p'| = |p|$. We do this by transforming every discrete coalescent tree on *p* into its extended ranked version. It remains to show that the resulting path is a valid path from $T_r$ to $R_r$ indeed, meaning that each pair $T'_r, R'_r$ of trees on $p'$ is connected by an ${\mathrm {RNNI}}$ move. We do this by considering every possible move between $T'$ and $R'$ on *p*, which can be an ${\mathrm {NNI}}$ move, a rank move, or a length move. Note that the tree $T'$ is identical to the subtree ${T'_r}^d$ of $T'_r$, and the same is true for $R'$ and ${R'_r}^d$.
  If an ${\mathrm {NNI}}$ move or rank move is performed on $T'$ to result in $R'$, the subtrees ${R'_r}^d$ and ${T'_r}^d$ are connected by exactly the same move. If the set ${\bar{S}} \subseteq \{1, \ldots , m\}$ is the set of times that are assigned to internal nodes in $T'$, then neither an ${\mathrm {NNI}}$ nor a rank move changes this set, meaning that internal nodes of $R'$ also are assigned elements of ${{\bar{S}}}$. Therefore, the set $S = \{1, \ldots , m\} \setminus {{\bar{S}}}$ is the set of times that are not assigned to internal nodes for both $T'$ and $R'$. With Algorithm 2 it follows that the caterpillar trees ${T'_r}^c$ and ${R'_r}^c$ are identical. We can conclude that $T'_r$ and $R'_r$ are neighbours in ${\mathrm {RNNI}}$, since $R'_r$ results from an ${\mathrm {RNNI}}$ move on $T'_r$, more specifically on the subtree ${T'_r}^d$.
  If there is a length move on *p* between $T'$ and $R'$, the time of an internal node in $T'$ is increased or decreased by one. Let *t* be the the time of that internal nodes that changes to $t+i$ for $i \in \{1, -1\}$. Note that the time cannot change to become $m+1$, as we consider the shortest path in ${\mathrm {DCT}}_m$. There is hence a node in $T'$ that has time *t*, but none with time $t+i$, while the node inducing the same cluster in $R'$ has time $t+i$ and no node with time *t* exists there. All other nodes of the trees $T'$ and $R'$ coincide. For the extended ranked version $T'_r$ of $T'$ this means that there is an internal node of rank $t+i$ in the subtree ${T'_r}^c$, as by the construction of the extended ranked version of a tree every integer in $\{1, \ldots , m\}$ is assigned as a time to an internal node in $T'_r$. Similarly, there is an internal node of rank *t* in ${R'_r}^c$, but none with rank $t + i$. All other nodes coincide in ${T'_r}^c$ and ${R'_r}^c$ and the difference between ${T'_r}^d$ and ${R'_r}^d$ is the same as between $T'$ and $R'$, that is, the time of one internal node that changes from *t* to $t+i$. We can conclude that there is a rank move between $T'_r$ and $R'_r$ swapping the ranks of the internal nodes of rank *t* and $t+i$ for $i \in \{1,-1\}$. An example of such a length move and the corresponding rank move is depicted in Fig. 4. $T'_r$ and $R'_r$ are hence ${\mathrm {RNNI}}$ neighbours, if $T'$ and $R'$ are connected by a length move.
  By replacing the moves on *p* between discrete coalescent trees by moves on $p'$ on ranked trees as described above, it follows that $p'$ is an ${\mathrm {RNNI}}$ path between the ranked trees $T_r$ and $R_r$ with length $|p'| = |p|$. With (Collienne and Gavryushkin 2021, Theorem 1) we know that ${\textsc {FindPath}}$ computes a shortest paths between the ranked trees $T_r$ and $R_r$ in ${\mathrm {RNNI}}$, and we can follow $|p| = |p'| \ge |{\mathrm {FP}}(T,R)|$.
2. $$|p| \le |{\mathrm {FP}}(T_r,R_r)|$$
  In this case we consider the path ${\mathrm {FP}}(T_r,R_r)$ in ${\mathrm {RNNI}}$ and transform this path to a path $p'$ between the discrete coalescent trees *T* and *R*. Therefore, we transform every tree on ${\mathrm {FP}}(T_r,R_r)$ into a discrete coalescent tree by just considering the subtrees induced by the set $\{a_1, \ldots , a_n\}$. We show that every pair of subsequent trees $T', R'$ on $p'$ is connected by a rank move, ${\mathrm {NNI}}$ move, or length move. It then follows that $p'$ is a valid path in ${\mathrm {DCT}}_m$ with length $|p'| = |{\mathrm {FP}}(T_r,R_r)|$. We now consider all possible ${\mathrm {RNNI}}$ moves between the ranked trees $T'_r$ and $R'_r$ on ${\mathrm {FP}}(T_r,R_r)$ and see how the corresponding discrete coalescent trees $T'$ and $R'$ are related.
  Note that by the construction of extended ranked versions $T'_r$ and $R'_r$ of discrete coalescent trees with Algorithm 2, all clusters in the added caterpillar subtrees ${T'_r}^c$ and ${R'_r}^c$ coincide. With Lemma 1 it follows that there cannot be a move on ${\mathrm {FP}}(T_r,R_r)$ that changed any of these clusters.
  If the move between $T'_r$ and $R'_r$ is an ${\mathrm {NNI}}$ move, it hence must be an ${\mathrm {NNI}}$ move in the subtree ${T'_r}^d$, as ${\mathrm {NNI}}$ moves involving nodes of the subtree ${T'_r}^c$ would result in changing a cluster. Since the subtree ${T'_r}^d$ is identical to $T'$ and ${R'_r}^d$ is identical to $R'$, it follows that $T'$ and $R'$ are connected by an ${\mathrm {NNI}}$ move.
  If the move between $T'_r$ and $R'_r$ is a rank move, it can either be a rank move between two nodes in ${T'_r}^d$ or between one node in ${T'_r}^d$ and one node in ${T'_r}^c$. Note that no rank move inside ${T'_r}^c$ is possible, as this subtree is a caterpillar tree. If the rank move is inside the subtree ${T'_r}^d$, the same move can happen in the discrete coalescent tree $T'$ and as for ${\mathrm {NNI}}$ moves, the rank move on $T'$ results in the discrete coalescent tree $R'$ that is neighbour of $T'$ on $p'$. We now consider a rank move swapping the rank *t* of a node in the subtree ${T'_r}^d$ with the rank $t+i$ of a node in ${T'_r}^c$, with $i \in \{1,-1\}$. The only difference between the subtrees ${T'_r}^d$ and ${R'_r}^d$, which are identical to $T'$ and $R'$, is the time of one internal node, which changes from *t* to $t+i$. Therefore, $T'$ and $R'$ are connected by a length move.
  We can hence construct a path $p'$ by translating every move on ${\mathrm {FP}}(T_r,R_r)$ to a move between discrete coalescent trees. These two paths have the same length, $|p'| = |{\mathrm {FP}}(T_r,R_r)|$. Since $p'$ is a path between the discrete coalescent trees *T* and *R* and *p* is, by assumption, a shortest path between these trees, it follows $|p| \le |p'| = |{\mathrm {FP}}(T_r,R_r)|$

$$\square $$

Part 2 of the proof of Theorem 1 gives a construction of a shortest path between two discrete coalescent trees *T* and *R*, using the shortest path ${\mathrm {FP}}(T_r,R_r)$ between the extended ranked versions of the given trees. We denote a path between discrete coalescent trees *T* and *R* that results from this construction by ${\mathrm {FP}}^+(T,R)$. Length moves on a tree $T'$ on such a path result from rank moves between a node in ${T'_r}^d$ and a node in ${T'_r}^c$ in $T'_r$. We therefore refer to these rank moves in $T'_r$ that induce length moves in $T'$ as *rank moves corresponding to length moves*.

We now introduce a modified version of ${\textsc {FindPath}}$, to be used for discrete coalescent trees in ${\mathrm {DCT}}_m$, without requiring the input trees to be transformed into ranked trees. This algorithm is called ${\textsc {FindPath}}^+$ (Algorithm 3) and computes a path ${\mathrm {FP}}^+(T,R)$ between two input trees *T* and *R*. We will see that this path is identical to the path resulting from restricting all trees on the path ${\mathrm {FP}}(T_r,R_r)$, computed by ${\textsc {FindPath}}$ between the extended ranked versions $T_r$ and $R_r$ of the input trees, to the subtrees induced by $\{a_1, \ldots , a_n\}$.

${\textsc {FindPath}}^+$ iterates through all times $k = 1, \ldots , m$ that internal nodes could have in *R* to construct a path *p*, initially starting with $p = [T]$. If in iteration *k*, *R* has an internal node with time *k* that induces a cluster *C*, the most recent common ancestor of *C* in the currently last tree $T_1$ on *p* is decreased by ${\mathrm {NNI}}$, rank, or length moves, until it reaches rank *k*. If on the other side there is no node with time *k*, we find for the lowest integer *i* that is bigger than *k* such that there is no internal node in $T_1$ that is assigned the time *i* (line 10 in Algorithm 3). Note that in this case, there is an internal node in the caterpillar subtree ${R_r}^c$ of the extended ranked version $R_r$ of *R* that has rank *k* and induces a cluster *C*. By the choice of *i* and the construction of extended ranked versions of discrete coalescent trees, the internal node in ${T_1}_r$ with rank *i* induces the same cluster *C* in ${T_1}_r$. The moves that would happen on ${\textsc {FindPath}}$ to get from ${T_1}_r$ to $R_r$ would now decrease the rank of $(C)_{T_1}$ from *i* to *k* by rank moves corresponding to length moves. Therefore, ${\textsc {FindPath}}^+$ increases the time of all internal nodes that have rank between *k* and *i* in $T_1$ by one, which requires length moves (line 11), ending in a tree that does not have an internal node with time *k*. Since every tree on ${\mathrm {FP}}^+(T,R)$ is the same as the tree at the same position on ${\mathrm {FP}}(T_r,R_r)$, restricted to the subtrees induced by $\{a_1, \ldots , a_n\}$, ${\textsc {FindPath}}^+$ computed shortest paths in ${\mathrm {DCT}}_m$.

Note that we do not need the parameter *m* in practice, as the distance between any two trees in ${\mathrm {DCT}}_{m'}$ is the same as their distance in ${\mathrm {DCT}}_m$ for any $m > m'$. This follows from ${\textsc {FindPath}}$ applied to extended ranked versions of trees *T* and *R*, where for $m>m'$ all clusters induced by nodes with rank greater than $m'$ are the same in *T* and *R*, meaning that they are preserved on ${\mathrm {FP}}(T_r,R_r)$. And since $d_{{\mathrm {DCT}}}(T,R) = |{\mathrm {FP}}(T_r,R_r)|$ (Theorem 1), it follows that the distances between *T* and *R* are the same for all $m > m'$. If the distance between two trees is to be computed, we can simply choose *m* to be the maximum root height of the given trees and compute their distance in ${\mathrm {DCT}}_m$.
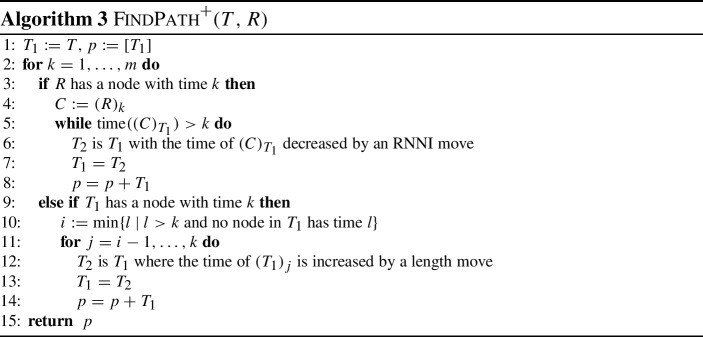


Note that the uniqueness of the moves that decrease or increases the time of internal nodes follows from the uniqueness of moves on ${\textsc {FindPath}}$ of ranked trees (Collienne and Gavryushkin 2021, Proposition 1). The worst-case running time of ${\textsc {FindPath}}^+$ on discrete coalescent trees is ${\mathcal {O}}(mn)$, which we will explain in detail in Sect. 4.3.

## Geometrical properties of ${\mathrm {DCT}}_m$

### Cluster property

A tree space has the *cluster property*, if all trees on every shortest path between two trees sharing a cluster *C* also contain *C*. This is a desirable property in evolutionary biology applications as trees sharing a cluster or subtree are expected to be closer to each other than to a tree not sharing a cluster with them. This property is also desirable in centroid-based summary methods, where a summary tree minimises a function on distances to trees in the given tree set. For a given sample of trees containing a common subtree, it is expected that their summary tree also contains this subtree. It is therefore desirable to have a tree space that has the cluster property. Related to the cluster property is the idea to split the computation of distances into computing the distance between the subtrees induced by a shared cluster and the remaining tree (Bordewich and Semple 2005).

A mathematical motivation for investigating the cluster property in ${\mathrm {RNNI}}$ is its importance in a similar tree space, the nearest neighbour interchange graph (${\mathrm {NNI}}$). In the ${\mathrm {NNI}}$ graph, trees have no times and ${\mathrm {NNI}}$ moves are allowed on every edge, while rank moves and length moves are not possible as no times are assigned to internal nodes. Computing the ${\mathrm {NNI}}$ distance between two trees is ${\mathcal {NP}}$-hard (Dasgupta et al. 2000), and the proof relies on the fact that this tree space does not have the cluster property (Li et al. 1996). In the ${\mathrm {RNNI}}$ graph, however, distances can be computed in polynomial time using ${\textsc {FindPath}}$ (Collienne and Gavryushkin 2021), which preserves common clusters (Lemma 1). The question whether ${\mathrm {RNNI}}$ has the cluster property is hence natural, and will be settled by Theorem 2.

#### Theorem 2

The ${\mathrm {RNNI}}$ graph has the cluster property.

#### Proof

**Fig. 5:**
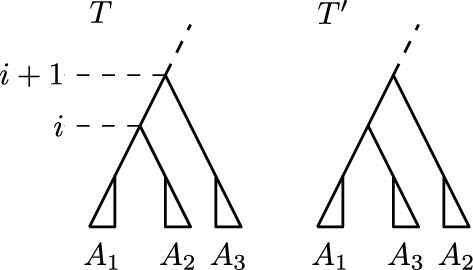
Tree *T* and ${\mathrm {NNI}}$ neighbour $T'$, such that the cluster $C = A_1 \cup A_2$ is not present in $T'$, but in *T*

We assume to the contrary that there are two ranked trees *T* and *R* sharing a cluster *C* and a shortest path *p* between these trees where *C* is not present in every tree. We furthermore assume that there is no pair of trees $T',R'$ with $d_{{\mathrm {RNNI}}}(T',R') < d_{{\mathrm {RNNI}}}(T,R)$ that shares a cluster $C'$ and is connected by a shortest path $p'$ that does not preserve $C'$. We hence say that *T* and *R* give a minimum counterexample. Because of this minimality assumption on the length of *p*, the first tree $T'$ following *T* on *p* does not contain *C*. Since ${\mathrm {NNI}}$ moves only change one cluster, *C* is the only cluster changed in $T'$ compared to *T*, and all nodes with rank below $(C)_T$ induce the same clusters in *T* and $T'$ (Fig. 5). We now compare distances $d_{{\mathrm {RNNI}}}(T,R)$ and $d_{{\mathrm {RNNI}}}(T',R)$ by using properties of ${\textsc {FindPath}}$.

Therefore, we first show that all nodes with rank less than *i* induce the same clusters in *T* and *R*. If this was not the case, then all trees on ${\mathrm {FP}}(R,T)$ and ${\mathrm {FP}}(R,T')$ coincide up to iteration $i = {\mathrm {rank}}((C)_T)$, in which the cluster considered on ${\mathrm {FP}}(R,T)$ is *C*. Let $R'$ denote the tree at this point of the path, meaning that ${\mathrm {FP}}(R,T)$ and ${\mathrm {FP}}(R,T')$ coincide up to this tree $R'$. Since ${\textsc {FindPath}}$ preserves clusters (Lemma 1), $R'$ has the cluster *C*. Furthermore, the tree $T'$, which does not have the cluster *C*, is on a shortest path from *T* to *R*. This is a contradiction to the minimality assumption on *T* and *R*, so we can assume that all clusters induced by nodes with rank less than *i* coincide in *R* and *T*.

We now show that by the minimality assumption on *T* and *R*, *C* is induced by the node of rank *i* in *R*. We therefore assume to the contrary that *C* is induced by a node of rank greater than *i* in *R*. Then the first cluster considered on ${\mathrm {FP}}(T,R)$ and ${\mathrm {FP}}(T',R)$ is a cluster *D* induced by $(R)_i$ that does not intersect *C*. By the definition of most recent common ancestors, both subtrees rooted in the children of the most recent common ancestor of *D* must contain elements of *D*. Therefore, the most recent common ancestor of *D* has rank greater than $i+1$ in both *T* and $T'$, as *D* would intersect *C* if $(D)_T$ or $(D)_{T'}$ had rank *i* or $i+1$ (see Fig. 5). If ${\mathrm {rank}}(D)_T > i+2$, then the first move on ${\mathrm {FP}}(T,R)$ and ${\mathrm {FP}}(T',R)$ applies the same changes to clusters in *T* and $T'$, resulting in trees $T_1$ and $T'_1$, respectively. Since $T_1$ has the cluster *C*, but $T'_1$ does not, this contradicts our assumption that *T* and *R* give a minimum counterexample. If ${\mathrm {rank}}(D)_T = i+2$, then the rank of the most recent common ancestor of *D* decreases from $i+2$ to *i* in the first two steps of ${\mathrm {FP}}(T,R)$ and ${\mathrm {FP}}(T',R)$, which result in trees $T_2$ and $T'_2$ that are ${\mathrm {NNI}}$ neighbours connected by the same move as *T* and $T'$. This again contradicts the minimality assumption on *T* and *R*. Hence there can be no such a cluster *D* in *R* and we can conclude that *C* is induced by the node of rank *i* in *R*.

The first iteration of ${\textsc {FindPath}}$ applied to the pair of trees $(T',R)$ hence considers the cluster *C*. To construct the cluster *C* in $T'$, there is just one ${\mathrm {NNI}}$ move needed, which results in the tree *T*, as *T* and $T'$ are ${\mathrm {NNI}}$ neighbours such that *T* contains *C* and $T'$ does not (Fig. 5). Therefore, *T* is the first tree following $T'$ on ${\mathrm {FP}}(T',R')$, resulting in $|{\mathrm {FP}}(T',R)| = |{\mathrm {FP}}(T',T)| + |{\mathrm {FP}}(T,R)|$ and hence $d_{{\mathrm {RNNI}}}(T',R) = 1 + d_{{\mathrm {RNNI}}}(T,R)$. From the assumption that $T'$ is the first tree on a shortest path from *T* to *R* we can however infer $d_{{\mathrm {RNNI}}}(T',R) = d_{{\mathrm {RNNI}}}(T,R) - 1$, which leads to a contradiction. There is hence no shortest path between *T* and *R* that does not preserve *C*, which proves the cluster property for ${\mathrm {RNNI}}$. $\square$

The fact that ${\textsc {FindPath}}^+$ computes shortest paths in ${\mathrm {DCT}}_m$ already suggests that shortest paths in ${\mathrm {RNNI}}$ and ${\mathrm {DCT}}_m$ have similar properties. Indeed, the cluster property in ${\mathrm {DCT}}_m$ follows from Theorem 2.

#### Corollary 1

The graph ${\mathrm {DCT}}_m$ has the cluster property.

#### Proof

Assume that there is a shortest path between two trees *T* and *R* in ${\mathrm {DCT}}_m$ that does not preserve a common cluster. This path corresponds to a path between $T_r$ and $R_r$, the extended ranked versions of *T* and *R* in ${\mathrm {RNNI}}$, as already discussed in Theorem 1. Since this path has the same length as the one between $T_r$ and $R_r$, it is a shortest path in ${\mathrm {RNNI}}$ as well, which leads to a contradiction to Theorem 2. $\square$

### Caterpillar trees

In this subsection we focus on the set of caterpillar trees and establish some properties of shortest paths between those trees in both ${\mathrm {RNNI}}$ and ${\mathrm {DCT}}_m$. In Theorem 3 we will see that, in both ${\mathrm {DCT}}_m$ and ${\mathrm {RNNI}}$, any two caterpillar trees are connected by a shortest path consisting only of caterpillar trees. We say that a set of trees is *convex* in a tree space, if there is a shortest path between any two trees in this set that stays within the set. The set of caterpillar trees is hence convex in ${\mathrm {RNNI}}$ and ${\mathrm {DCT}}_m$. The ${\mathrm {NNI}}$ space of unranked trees however does not have this property (Gavryushkin et al. 2018). Based on the convexity of the set of caterpillar trees in ${\mathrm {RNNI}}$ we introduce a way to compute distances between caterpillar trees in this space in time ${\mathcal {O}}(n \sqrt{\log n})$ in Corollary 2, and hence with better worst-case time complexity than ${\textsc {FindPath}}$. Whether this complexity can be achieved in ${\mathrm {DCT}}_m$ for pairs of caterpillar trees is an open question.

#### Theorem 3

The set of caterpillar trees is convex in ${\mathrm {DCT}}_m$.

#### Proof

Let *T* and *R* be two caterpillar trees in ${\mathrm {DCT}}_m$. We prove the theorem by showing that there is a caterpillar tree $T'$ that is a neighbour of *T* and closer to *R* than *T*, that is, $d_{{\mathrm {DCT}}_m}(T', R) = d_{{\mathrm {DCT}}_m}(T, R) - 1$. The existence of a shortest path consisting only of caterpillar trees between *T* and *R* follows from this property inductively. In the proof of Theorem 1 we have seen that all paths in ${\mathrm {DCT}}_m$ can be transformed to paths in ${\mathrm {RNNI}}$ between the extended ranked versions of trees, and vice versa, such that these two paths are of equal length. It is therefore sufficient to show that for trees $T_r$ and $R_r$, the extended ranked versions of *T* and *R*, there is a tree $T'$ that is a neighbour of *T* with extended ranked version $T'_r$ such that ${T'_r}^d$ is a caterpillar tree.

Let $a_k$ be the leaf with parent of highest rank in $R_r$ that is not at the same position in $R_r$ as in $T_r$: $a_k := {{\,\mathrm{argmax}\,}}_{a_1, \ldots , a_n}\{{\mathrm {rank}}(a_i)_{R_r} \ |\ {\mathrm {rank}}(a_i)_{R_r} \ne {\mathrm {rank}}(a_i)_{T_r}\}$. You could also think of comparing the trees $T_r$ and $R_r$ in a top-down approach, starting at the root, and finding the first node that does not induce the same cluster in these two trees. Since all subtrees ${T_r}^d, {T_r}^c, {R_r}^d$ and ${R_r}^c$ are caterpillar trees, this node has a child that is a leaf, which is $a_k$. Let furthermore $a_j \in \{a_1, \ldots , a_{m+2}\}$ be the leaf directly ‘above’ $a_k$ in $T_r$, i.e. ${\mathrm {rank}}(a_j)_{T_r} = {\mathrm {rank}}(a_k)_{T_r} + 1$. Note that with the definition of $a_k$ it immediately follows that $a_j$ is ‘below’ $a_k$ in $R_r$ (${\mathrm {rank}}(a_j)_{R_r} < {\mathrm {rank}}(a_k)_{R_r}$). If otherwise it was ${\mathrm {rank}}(a_j)_{R_r} > {\mathrm {rank}}(a_k)_{R_r}$, the parent of $a_j$ would have the same rank in $T_r$ as in $R_r$ and ${\mathrm {rank}}(a_j)_{T_r} > {\mathrm {rank}}(a_k)_{T_r}$ would follow, which contradict our choice of $a_j$.

**Fig. 6:**
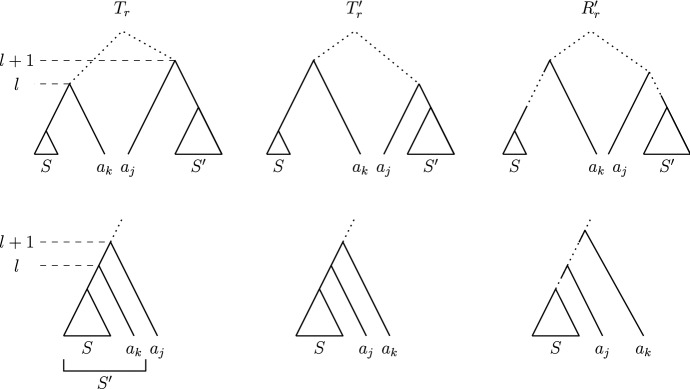
The two possible versions of trees $T_r$ (left), $T'_r$ (middle), and $R'_r$ as described in the proof of Theorem 3. Between $T_r$ and $T'_r$ only the ranks of the parents of $a_j$ and $a_k$ are exchanged, the rest of the trees coincide. At the bottom the case that $(a_j)_T$ is parent of $(a_k)_T$ and $S' = S \cup \{a_k\}$ is displayed. $S'$ is a cluster in all three trees at the bottom. At the top $(a_j)_T$ and $(a_k)_T$ are in the two different subtrees $T_r^d$ and $T_r^c$ (the same in $T'_r$ and $R'_r$), which is also true for the disjoint sets *S* and $S'$, which are present as clusters in all three trees. Dotted lines represent remaining parts of trees, which are equal in $T_r$ and $T'_r$, but different to $R'_r$. Note that the rank difference of $(a_k)_{R'_r}$ and $(a_j)_{R'_r}$ does not need to be one, which it is in $T_r$ and $T'_r$

Let $T'_r$ be the tree resulting from $T_r$ by an ${\mathrm {NNI}}$ move or rank move exchanging the ranks of $(a_k)_{T_r}$ and $(a_j)_{T_r}$. An ${\mathrm {NNI}}$ move is necessary if these two nodes are connected by an edge, otherwise a rank move corresponding to a length move is performed on $T_r$ to obtain $T'_r$ (Fig. 6). ${T'_r}^d$ is a caterpillar tree in both cases. We will use properties of shortest paths computed by ${\textsc {FindPath}}$ to show that $|{\mathrm {FP}}(R_r,T'_r)| = |{\mathrm {FP}}(R_r,T_r)| - 1$.

Since all clusters of $T_r$ and $T'_r$ induced by nodes of rank less than ${\mathrm {rank}}(a_k)_{T_r}$ coincide, the paths ${\mathrm {FP}}(R_r,T_r)$ and ${\mathrm {FP}}(R_r,T'_r)$ coincide up to a ranked tree $R'_r$, which contains all these clusters. We therefore compare only the lengths of ${\mathrm {FP}}(R'_r,T_r)$ and ${\mathrm {FP}}(R'_r,T'_r)$. From ${\mathrm {rank}}(a_j)_{R_r} < {\mathrm {rank}}(a_k)_{R_r}$ we can follow ${\mathrm {rank}}(a_j)_{R'_r} < {\mathrm {rank}}(a_k)_{R'_r}$, as $a_j$ and $a_k$ are not in any of the clusters considered by ${\textsc {FindPath}}$ before $R'_r$, which means that their parents do not exchange ranks before $R'_r$. We now consider the move on ${\mathrm {FP}}(R_,T_r)$ on the tree $R'_r$, which corresponds to some iteration *l* in ${\textsc {FindPath}}$. Note that by the choice of $R'_r$, all clusters with rank less than $rank(a_k)_{T_r}$ coincide between $R'_r$ and $T_r$, from which we can follow $l = rank(a_k)_{T_r}$.

By our assumptions on $T_r$ consisting of two caterpillar trees joined at the root, the cluster considered in iteration *l* is $S \cup \{a_k\}$, where *S* is a cluster that is present in all three trees $T_r, T'_r,$ and $R_r$. In the following iteration $l+1 = {\mathrm {rank}}(a_j)_{T_r}$, $S' \cup \{a_j\}$ is considered for a cluster $S'$. $S'$ either equals $S \cup \{a_k\}$, if $T_r$ and $T'_r$ are connected by an ${\mathrm {NNI}}$ move (bottom of Fig. 6), or $S'$ is a cluster present in ${T_r}^c$, ${T'_r}^c$, and ${R'_r}^c$, if $T_r$ and $T'_r$ are connected by a rank move (top of Fig. 6). Decreasing the rank of $(S \cup \{a_k\})_{R'_r}$ takes ${\mathrm {rank}}(S \cup \{a_k\})_{R'_r} - l$ ${\mathrm {RNNI}}$ moves in both cases. Because the rank of $(S \cup \{a_j\})_{R'_r}$ increases by one when the parents of $a_k$ and $a_j$ swap ranks in this iteration, the following iteration for $S' \cup \{a_j\}$ needs ${\mathrm {rank}}(S' \cup \{a_j\})_{R'_r} + 1 - (l+1)$ ${\mathrm {RNNI}}$ moves. On ${\mathrm {FP}}(R_r,T'_r)$ however, first ${\mathrm {rank}}(S' \cup \{a_j\})_{R'_r} - l$ ${\mathrm {RNNI}}$ moves decrease the rank of $(S' \cup \{a_j\})_{R'_r}$ in $R'_r$, and then ${\mathrm {rank}}(S \cup \{a_k\})_{R'_r} - (l+1)$ are needed for $S \cup \{a_k\}$. In total, these two iterations combined result in at least one extra move on ${\mathrm {FP}}(R_r, T_r)$ comparing to ${\mathrm {FP}}(R_r, T'_r)$.

The only difference in the trees after iteration $l+1$ on the two different paths is the order of ranks of the parents of $a_j$ and $a_k$. Since the rest of $T_r$ and $T'_r$ coincide, the remaining parts of ${\mathrm {FP}}(R_r, T_r)$ and ${\mathrm {FP}}(R_r, T'_r)$ consist of the same moves. With our previous observation we can follow $d_{{\mathrm {RNNI}}}(R_r,T_r) = d_{{\mathrm {RNNI}}}(R_r,T'_r) + 1$, and hence $T'_r$ is on a shortest path from $T_r$ to $R_r$. $\square$

Note that with ${\mathrm {RNNI}}= {\mathrm {DCT}}_{n-1}$ it follows that the set of caterpillar trees is convex in ${\mathrm {RNNI}}$. This convexity property implies that the distance between caterpillar trees can be computed more efficiently than by ${\textsc {FindPath}}$. We prove this in the rest of this section. To do so, we first establish that the problem of computing a shortest path consisting only of caterpillar trees can be interpreted in a few different ways.

A problem related to the shortest path problem for caterpillar trees in ${\mathrm {RNNI}}$ is the *Token Swapping Problem* (Kawahara et al. 2017) on a special class of graphs, so-called lollipop graphs. We will show that a pair of caterpillar trees in ${\mathrm {RNNI}}$ can be translated to an instance of the Token Swapping Problem, such that the ${\mathrm {RNNI}}$ distance between the trees is equal to the number of swaps, as explained in the following. An instance of the token swapping problem is a simple graph where every vertex is assigned a token. Two tokens are allowed to swap positions if they are on vertices that are connected by an edge. Each token is assigned a unique goal vertex, and the aim is to find the minimum number of token swaps for all tokens to reach their goal vertex.

**Fig. 7:**
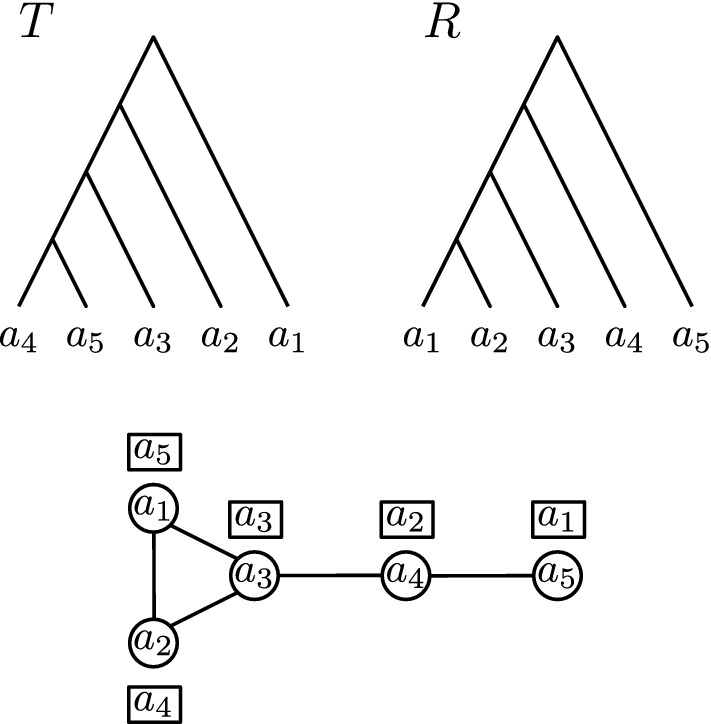
Two caterpillar trees *T* and *R* and the corresponding instance of the token swapping problem. Vertex labels are in circles and token goal vertices in rectangles

The problems of computing distances between caterpillar trees can be seen as an instance of the token swapping problem on lollipop graphs. A lollipop graph is a graph consisting of a complete graph that is connected to a path by exactly one edge. An instance of the token swapping problem that corresponds to the distance problem for caterpillar trees is described in the following. An example is illustrated in Fig. 7. Let *T* and *R* be caterpillar trees with

$$\begin{aligned} {\mathrm {rank}}(a_1)_R= & {} {\mathrm {rank}}(a_2)_R< {\mathrm {rank}}(a_3)_R< \ldots< {\mathrm {rank}}(a_n)_R \text { and}\\ {\mathrm {rank}}(b_1)_T= & {} {\mathrm {rank}}(b_2)_T< {\mathrm {rank}}(b_3)_T< \ldots < {\mathrm {rank}}(b_n)_T \end{aligned}$$

such that $[b_1, \ldots , b_n]$ is a permutation of $[a_1, \ldots , a_n]$. The corresponding instance of the token swapping problem consists of a lollipop graph consisting of a complete graph on three leaves, connected to a path of length $n-3$ by an edge. The vertex in the complete graph incident to the edge connecting the complete graph with the path is labelled by $a_3$, the other ones in the complete graph are labelled by $a_1$ and $a_2$. The vertices on the paths are then labelled inductively, starting at the neighbour of $a_3$, such that the unique unlabelled neighbour of the last already labelled node with label $a_{i-1}$ is labelled by $a_i$. We place the token on vertex $a_i$ that has $b_i$ as goal vertex for all $i \ge 3$. On $a_1$ and $a_2$, which represent the cherry of the caterpillar tree, we place the tokens with goal vertices $b_1$ and $b_2$ so that if $a_i = b_j$ for some $i,j \in \{1,2\}$, the token with goal vertex $b_j=a_i$ is placed on the node labelled $a_i=b_j$. Since the only moves between two caterpillar trees in ${\mathrm {RNNI}}$ are ${\mathrm {NNI}}$ moves, which simply swap two leaves, they correspond to swapping two tokens in the above described instance of the token swapping problem.

Therefore, the algorithm described by Kawahara et al. (2017) to solve the token swapping problem on lollipop graphs can be used for computing distances between caterpillar trees. It however has worst-case time complexity ${\mathcal {O}}(n^2)$, the same as ${\textsc {FindPath}}$.

In the following we present an algorithm for computing distances between caterpillar trees with better worst-case time complexity, ${\mathcal {O}}(n \sqrt{\log n})$, for ${\mathrm {RNNI}}$ (Corollary 2). To do so, we first establish a formula to express distances between two caterpillar trees in ${\mathrm {RNNI}}$ (Theorem 4). This algorithm can also be used to solve the token swapping problem on lollipop graphs, improving the worst-case running time of the known algorithm (Kawahara et al. 2017).

For improving on the time-complexity of computing distances between caterpillar trees, we use a representation of caterpillar trees as a list of leaves, ordered according to increasing rank of their parents. The caterpillar tree on the left of Fig. 7 for example can be represented as

$$\begin{aligned} \text { or }[a_5,a_4,a_3,a_2,a_1].\end{aligned}$$

There are two possible list representations of a caterpillar tree because the first two leaves ($a_4$ and $a_5$ in this example) share their parent of rank one. For two given caterpillar trees *T* and *R* we call a pair of leaves $(a_i,a_j)$ *transposition* in *T* with respect to *R*, if the rank of the parent of $a_i$ is lower than the rank of the parent of $a_j$ in *T*, and the opposite is true for *R*: ${\mathrm {rank}}(a_i)_T < {\mathrm {rank}}(a_j)_T$ and ${\mathrm {rank}}(a_i)_R > {\mathrm {rank}}(a_j)_R$. For two leaves $a_i$ and $a_j$ in a caterpillar tree *T* we say that $a_i$ is *below* $a_j$ and $a_j$ is *above* $a_i$ in *T* if ${\mathrm {rank}}(a_i)_T < {\mathrm {rank}}(a_j)_T$.

#### Theorem 4

Let *T* and *R* be caterpillar trees in ${\mathrm {RNNI}}$ such that

$$\begin{aligned} 1 = {\mathrm {rank}}(a_1)_R = {\mathrm {rank}}(a_2)_R< {\mathrm {rank}}(a_3)_R< \ldots < {\mathrm {rank}}(a_n)_R = n-1. \end{aligned}$$

Define

$$\begin{aligned} P(T,R)= & {} \{(a_i,a_j)\ |\ {\mathrm {rank}}(a_i)_T< {\mathrm {rank}}(a_j)_T \text { and } {\mathrm {rank}}(a_i)_R> {\mathrm {rank}}(a_j)_R\}, \\ M(T,R)= & {} \{a_i\ |\ \text {for all } l \text { with } {\mathrm {rank}}(a_l)_T \le {\mathrm {rank}}(a_i)_T \text { it is } {\mathrm {rank}}(a_l)_R > {\mathrm {rank}}(a_i)_R\} \\&\cap \{a_i \ |\ {\mathrm {rank}}(a_i)_T < \min \{{\mathrm {rank}}(a_1)_T, {\mathrm {rank}}(a_2)_T\}\}. \end{aligned}$$

Then for ${m(T,R) = |M(T,R)|}$ and ${p(T,R) = |P(T,R)|}$:

$$\begin{aligned} d_{{\mathrm {RNNI}}}(T,R) = p(T,R) - m(T,R). \end{aligned}$$

The set *P*(*T*, *R*) in Theorem 4 is the set of transpositions for the caterpillar tree *T* with respect to *R*. *M*(*T*, *R*) contains the leaves $a_i$ in *T* for which in the representation of *T* as a list (i) every leaf that is below $a_i$ in *T* (if $a_i$ is in the cherry, this includes the other cherry leaf) is strictly above $a_i$ in *R* and (ii) no cherry leaf of *R* is below $a_i$ in *T*. The caterpillar trees *T* and *R* in Fig. 7 for example have $P(T,R) = \{(a_1,a_3),(a_1,a_4),(a_1,a_5),(a_2,a_3),(a_2,a_4),(a_2,a_5),(a_3,a_4),(a_3,a_5)\}$ and $M(T,R) = \{a_3,a_4\}$.

#### Proof

Let *T* and *R* be caterpillar trees in ${\mathrm {RNNI}}$ as described above and let ${\widehat{d}}(T,R) := p(T,R) - m(T,R)$. For proving ${\widehat{d}}(T,R) = d_{{\mathrm {RNNI}}}(T,R)$ it is sufficient to show that there is a caterpillar tree $T'$ that is neighbour of *T* such that ${\widehat{d}}(T',R) = {\widehat{d}}(T,R) - 1$. Then it follows inductively that ${\widehat{d}}(T,R) = d_{{\mathrm {RNNI}}}(T,R)$, because the sets *P*(*T*, *T*) and *M*(*T*, *T*) are empty.

We assume, similar to the proof of Theorem 3 that *T* and *R* are caterpillar trees such that $a_k$ is the leaf with parent of highest rank in *R* that is not at the same position in *T*: $a_k := {{\,\mathrm{argmax}\,}}_{a_1, \ldots , a_n}\{{\mathrm {rank}}(a_i)_{R} \ |\ {\mathrm {rank}}(a_i)_{R} \ne {\mathrm {rank}}(a_i)_{T}\}$. Let $T'$ be the tree that results from an ${\mathrm {NNI}}$ move on *T* swapping the leaves $a_k$ and $a_i$ with ${\mathrm {rank}}(a_i)_T = {\mathrm {rank}}(a_k)_T + 1$. In the proof of Theorem 3 we saw that it follows $d_{{\mathrm {RNNI}}}(T',R) = d_{{\mathrm {RNNI}}}(T,R) - 1$. We now prove ${\widehat{d}}(T',R) = {\widehat{d}}(T,R) - 1$.

Therefore, we distinguish two cases: (i) ${\mathrm {rank}}(a_i)_T>1$ and (ii) ${\mathrm {rank}}(a_i)_T = 1$, meaning that $a_i$ is in the cherry of *T*.

**Fig. 8:**
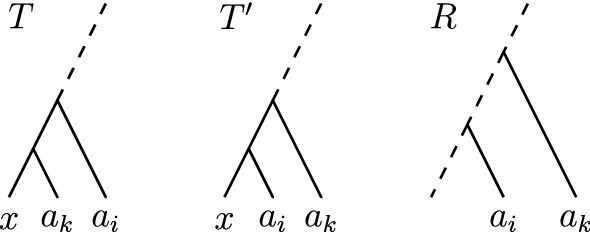
The caterpillar trees $T,T'$, and *R* as described in the proof of Theorem 4. *T* and $T'$ are neighbours and the dashed part of these trees coincide

- **Case (i)** By the definition of $a_k$, $(a_k,a_i)$ is a transposition in the set *P*(*T*, *R*). As $a_k$ and $a_i$ are the only leaves whose order changed between *T* and $T'$, they build the only transposition that is in *T* but not in $T'$ with respect to *R*. Hence it is $p(T',R) = p(T,R) - 1$. Because the definition of $a_k$ requires all leaves that are above $a_k$ in *R* to be at the same position in *T*, there is no leaf that is below $a_k$ in *T* and above it in *R*. Therefore, it is $a_k \notin M(T,R)$ and $a_k \notin M(T',R)$ for the same reason. If $a_i \in M(T,R)$, it follows $a_i \in M(T',R)$, as only the relationship between $a_i$ and $a_k$ changes and the inequalities required for $a_i$ to be in *M*(*T*, *R*) are true for $a_k$. For the same reason, if $a_i \notin M(T,R)$, it is $a_i \notin M(T',R)$. We can conclude $M(T',R) = M(T,R)$ and hence:
  $$\begin{aligned} {\widehat{d}}(T',R) = p(T',R) - m(T',R) = p(T,R) - 1 - m(T,R) = {\widehat{d}}(T,R) - 1 \end{aligned}$$
- **Case (ii)** As in the previous case, $(a_k,a_i)$ is a transposition in *P*(*T*, *R*), but not in $P(T',R)$. There is however another transposition that could be in *P*(*T*, *R*), but not in $P(T',R)$, that is the pair $(x,a_i)$, where *x* is the second cherry leaf of *T* (see Fig. 8). We now distinguish the case (a) that $(x,a_i)$ is not a transposition in *T* from the case (b) that $(x,a_i)$ is a transposition in *T* with respect to *R*.
  If $(x,a_i)$ is not a transposition in *T*, it follows $p(T',R) = p(T,R) - 1$, as $(a_k,a_i)$ is the only transposition that is in *P*(*T*, *R*), but not in $P(T',R)$. As in the previous case (i) it also follows $m(T,R) = m(T',R)$ and we conclude that it is
  $$\begin{aligned} {\widehat{d}}(T',R) = p(T',R) - m(T',R) = p(T,R) - 1 - m(T,R) = {\widehat{d}}(T,R) - 1 \end{aligned}$$
  If $(x,a_i)$ is a transposition, it is $p(T',R) = p(T,R) - 2$. To compare *m*(*T*, *R*) with $m(T',R)$, it is sufficient to consider the membership of $x,a_i,$ and $a_k$ in *M*(*T*, *R*) and $M(T',R)$. The relationships between all other leaves are the same in *M*(*T*, *R*) and $M(T',R)$, resulting in $a_j \in M(T,R)$ if and only if $a_j \in M(T',R)$ for all $j \in \{1, \ldots ,n \} \setminus \{a_i, a_k, x\}$. We again know by the definition of $a_k$ that $a_k \notin M(T,R)$ and $a_k \notin M(T',R)$. Since both *x* and $a_k$ are below $a_i$ in *T*, but above it in *R*, and neither of *x* and $a_k$ is in the cherry of *R*, it follows $a_i \in M(T,R)$, and similarly $a_i \in M(T',R)$. The leaf *x* is in *M*(*T*, *R*), as there is only one leaf $a_k$ that fulfils ${\mathrm {rank}}(a_k)_T \le {\mathrm {rank}}(x)_T$, and it also is ${\mathrm {rank}}(a_k)_R > {\mathrm {rank}}(a_k)_R$. Since $(x,a_i)$ is a transposition in *T*, it follows ${\mathrm {rank}}(x)_R < {\mathrm {rank}}(a_i)_R$. Together with ${\mathrm {rank}}(a_i)_{T'} = {\mathrm {rank}}(x)_{T'}$ it follows that $x \notin M(T',R)$. Therefore, it is $m(T',R) = m(T,R) - 1$ and we can conclude that in total
  $$\begin{aligned} {\widehat{d}}(T',R) \!= \!p(T',R) - m(T',R)\! = \!p(T,R) - 2 - (m(T,R)-1) \!=\! {\widehat{d}}(T,R) - 1. \end{aligned}$$

$$\square $$

#### Corollary 2

The distance between two caterpillar trees can be computed in ${\mathcal {O}}(n \sqrt{\log n})$ in ${\mathrm {RNNI}}$.

#### Proof

By Theorem 4 the distance between two caterpillar trees in ${\mathrm {RNNI}}$ is the number of transpositions between two sequences of length *n* minus *m*(*T*, *R*) as defined in Theorem 4. The value *m*(*T*, *R*) can be computed in time linear in *n* for any caterpillar tree *T* by considering the leaves of the tree ordered according to increasing rank of their parents. The number of transpositions of a sequence of length *n* (Kendall-tau distance) can be computed in time ${\mathcal {O}}(n \sqrt{\log n})$ (Chan and Pătraşcu 2010). This number is equal to *p*(*T*, *R*), as defined in Theorem 4, when ignoring transpositions for the pairs of leaves sharing a parent in *T* and *R*, respectively. The worst-case running time for computing the ${\mathrm {RNNI}}$ distance between caterpillar trees is therefore ${\mathcal {O}}(n \sqrt{\log n})$. $\square$

### Diameter and radius

In this section we investigate the *diameter* of ${\mathrm {RNNI}}$ and ${\mathrm {DCT}}_m$, which is the greatest distance between any pair of trees in each of these graphs, respectively, i.e. $\max \limits _{\text {trees }T,R}d(T,R)$. We first establish the exact diameter of ${\mathrm {RNNI}}$, improving the upper bound $n^2 - 3n - \frac{5}{8}$ given by Gavryushkin et al. (2018). Afterwards, we generalise this result to ${\mathrm {DCT}}_m$.

#### Theorem 5

The diameter of ${\mathrm {RNNI}}$ is $\frac{(n-1)(n-2)}{2}$.

#### Proof

For proving this theorem we use the fact that ${\textsc {FindPath}}$ computes shortest paths in ${\mathrm {RNNI}}$. Each iteration *i* of ${\textsc {FindPath}}$, applied to two ranked trees *T* and *R*, decreases the rank of the most recent common ancestor of a cluster *C*, induced by the node of rank *i* in *R*, in the currently last tree $T'$ on the already computed path (starting wth $T' = T$). The maximum rank of $(C)_{T'}$ at the beginning of iteration *i* is $n-1$, the rank of the root. As every move decreases the rank of $(C)_{T'}$ by one, there are at most $n-1-i$ moves in iteration *i*. The maximum length of a shortest path in ${\mathrm {RNNI}}$ is hence $\sum \limits _{i = 1}^{n-1} i = \frac{(n-1)(n-2)}{2}$. Note that the caterpillar trees $[\{a_1, a_2\}, \{a_1, a_2, a_3\}, \ldots , \{a_1, \ldots , a_n\}]$ and $[\{a_n, a_{n-1}\}, \{a_n, a_{n-1}, a_{n-2}\}, \ldots , \{a_n, \ldots , a_1\}]$ provide an example of trees that have distance $\frac{(n-1)(n-2)}{2}$, as already pointed out in Collienne and Gavryushkin (2021, Corollary 1), proving that this upper bound for the length of a shortest path is tight. $\square$

#### Theorem 6

The diameter of ${\mathrm {DCT}}_m$ is $\frac{(n-1)(n-2)}{2} + (m-n+1)(n-1)$.

#### Proof

In order to prove the diameter of ${\mathrm {DCT}}_m$, we consider the maximum number of moves that ${\textsc {FindPath}}$ can perform on the extended ranked versions $T_r$ and $R_r$ of any two trees *T* and *R*. With Theorem 1 it follows that this is the diameter of ${\mathrm {DCT}}_m$, indeed. Therefore, we distinguish ${\mathrm {RNNI}}$ moves in the subtrees on the leaf set $\{a_1, \ldots , a_n\}$ from the rank moves corresponding to length moves, i.e. rank moves between one node of each of the subtrees on leaf subsets $\{a_1, \ldots , a_n\}$ and $\{a_{n+1}, \ldots a_{m+2}\}$.

The maximum number of ${\mathrm {RNNI}}$ moves (excluding rank moves corresponding to length moves) on ${\mathrm {FP}}(T_r,R_r)$ follows from Theorem 5 and is $\frac{(n-1)(n-2)}{2}$. The maximum number of rank moves corresponding to length moves on a shortest path between $T_r$ and $R_r$ is reached when every internal node of the subtree $T_r^c$ of $T_r$ swaps rank with every internal node of the subtree $T_r^d$. The maximum number of such rank swaps corresponding to length moves is hence $(m-n+1)(n-1)$.

**Fig. 9:**
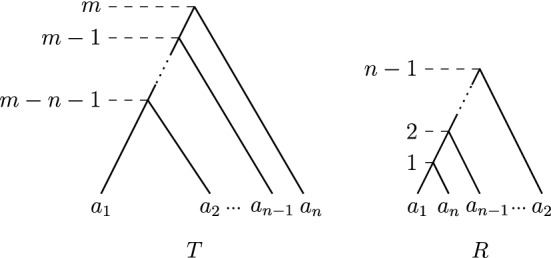
Trees *T* and *R* with distance $\frac{(n-1)(n-2)}{2} + (m-n+1)(n-1)$ as described in the proof of Theorem 6

The sum of the maximum number for ${\mathrm {RNNI}}$ and length moves is therefore $\frac{(n-1)(n-2)}{2} + (m-n+1)(n-1)$. To show that this upper bound is actually the diameter of ${\mathrm {DCT}}_m$ we give an example of trees *T* and *R* (Fig. 9) for which the path computed by ${\textsc {FindPath}}^+$ has length $\frac{(n-1)(n-2)}{2} + (m-n+1)(n-1)$. Both *T* and *R* are caterpillar trees defined as follows.

$$\begin{aligned}m-n-1 = {\mathrm {rank}}(a_1)_T = {\mathrm {rank}}(a_2)_T< {\mathrm {rank}}(a_3)_T< \ldots < {\mathrm {rank}}(a_n)_T = m\end{aligned}$$

and

$$\begin{aligned}1 = {\mathrm {rank}}(a_1)_R = {\mathrm {rank}}(a_n)_R< {\mathrm {rank}}(a_{n-1})_R< \ldots < {\mathrm {rank}}(a_1)_R = n-1.\end{aligned}$$

$$\square $$

Note that the worst-case running time of ${\textsc {FindPath}}$ in ${\mathrm {RNNI}}$ is ${\mathcal {O}}(n^2)$ and the running time of ${\textsc {FindPath}}^+$ in ${\mathrm {DCT}}_m$ is ${\mathcal {O}}(nm)$, as it depends on the diameter of the corresponding tree spaces. For computing a shortest path, there are no algorithm with better worst-case running time than these, as the running time for algorithms computing shortest paths is bounded from below by the diameter of the corresponding space. There can however be more efficient algorithms for computing distances, if this is not done by computing the shortest path as ${\textsc {FindPath}}$ and ${\textsc {FindPath}}^+$ do, but by finding an invariant that determines the distance without needing to compute every tree on a shortest path.

The *radius* of a graph is defined as the minimum distance of any vertex in the graph to the vertex with maximum distance from it, that is, $\min \limits _{T}\max \limits _{R} d(T,R)$, where *d* is the distance measure in the corresponding graph. In the following we see that the radius of ${\mathrm {RNNI}}$ equals its diameter, which is not true for ${\mathrm {DCT}}_m$, as we will see afterwards.

#### Theorem 7

The radius of ${\mathrm {RNNI}}$ equals its diameter $\frac{(n-1)(n-2)}{2}$.

#### Proof

We prove this theorem by showing that every ranked tree *T* in ${\mathrm {RNNI}}$ has a caterpillar tree *R* with distance $\frac{(n-1)(n-2)}{2}$ to *T*, using induction on the number of leaves *n*.

The base case $n=3$ is trivial, as all three trees in this space are caterpillar trees with distance one from each other. For the induction step we consider an arbitrary tree *T* with $n+1$ leaves. Let *x* and *y* be the leaves of *T* that share the internal node of rank one as parent in *T*, and let $T_n$ be the tree on *n* leaves resulting from deleting one of these leaves, say *x*, from *T*, and suppressing the resulting degree-2 vertex. By the induction hypothesis there is a caterpillar tree $R_n$ with distance $\frac{(n-1)(n-2)}{2}$ to $T_n$. Now consider the tree *R* resulting from adding *x* at the top of $R_n$ such that the root of *R* has *x* and $R_n$ as children.

**Fig. 10:**
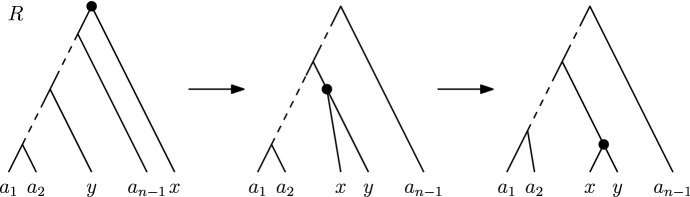
Initial $n - 1$ ${\mathrm {RNNI}}$ moves of ${\mathrm {FP}}(R,T)$ as described in the proof of Theorem 7. Removing the leaf *x* and suppressing the non-root node of degree two from the tree on the right results in $R_n$ as described in the theorem

We now consider ${\mathrm {FP}}(R,T)$. In the first iteration of ${\textsc {FindPath}}$, $(\{x,y\})_R$ moves down until it reaches rank one. Therefore, first $(x)_R$ moves down by ${\mathrm {NNI}}$ moves until it reaches rank ${\mathrm {rank}}(y)_R + 1$. Then a further ${\mathrm {NNI}}$ move creates an internal node with children *x* and *y*, before this node is moved down by rank swaps to reach rank one as depicted in Fig. 10. Altogether, there are $n-1$ ${\mathrm {RNNI}}$ moves needed in the first iteration, as the rank of the parent of *x* decreases by one within every move, starting at the root with rank *n* and ending at the internal node of rank one. The tree at the end of this first iteration on ${\mathrm {FP}}(R,T)$ is identical to $R_n$ when removing the leaf *x* and suppressing its parent (the node of rank one). Since the cluster $\{x,y\}$ is not considered again in ${\textsc {FindPath}}$, the remaining part of ${\mathrm {FP}}(R,T)$ contains the same moves as ${\mathrm {FP}}(R_n,T_n)$, and hence $|{\mathrm {FP}}(R,T)| = |{\mathrm {FP}}(R_n,R_n)| + n-1$. Therefore it is $d_{{\mathrm {RNNI}}}(T,R) = \frac{(n-1)(n-2)}{2} + n-1 = \frac{n(n-1)}{2}$, which proves the lemma. $\square$

**Fig. 11:**
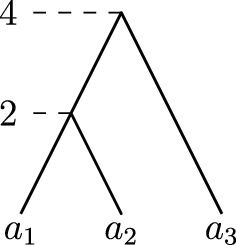
Tree in ${\mathrm {DCT}}_4$ on three leaves for which there is no tree at the diameter distance $5 = \frac{(n-1)(n-2)}{2} + (m-n+1)(n-1)$ from it

Unlike in ${\mathrm {RNNI}}$, the radius of ${\mathrm {DCT}}_m$ does not equal its diameter. A counterexample is given by the tree depicted in Fig. 11 on three leaves in ${\mathrm {DCT}}_4$. The diameter of ${\mathrm {DCT}}_4$ on three leaves is $\frac{(n-1)(n-2)}{2} + (m-n+1)(n-1) = 5$, but there is no tree with this distance from the tree provided in Fig. 11. The maximum distance between any tree in ${\mathrm {DCT}}_4$ and the tree in Fig. 11 is 4.

## Conclusion and future research questions

In this paper we introduced and analysed properties of the space of discrete coalescent trees ${\mathrm {DCT}}_m$. An important tool for establishing these characteristics of the tree space is the algorithm ${\textsc {FindPath}}$, which has been introduced by Collienne and Gavryushkin (2021) for ${\mathrm {RNNI}}$. We showed in Theorem 1 that ${\textsc {FindPath}}$ can also be used to solve the shortest path problem in ${\mathrm {DCT}}_m$. Therefore, it is required to transform discrete coalescent trees into ranked trees. With ${\textsc {FindPath}}^+$ we provided a modified version of the algorithm to avoid this conversion of trees. Afterwards, we established properties of ${\mathrm {DCT}}_m$ and ${\mathrm {RNNI}}$ such as the cluster property (Sect. 4.1), the convexity of the set of caterpillar trees (Sect. 4.2), diameter, and radius (Sect. 4.3). With the convexity of the set of caterpillar trees in ${\mathrm {RNNI}}$ we also found a more efficient way of computing distances between such trees, using the correspondence between caterpillar trees and permutations.

The worst-case time complexity of ${\textsc {FindPath}}^+$ for computing a shortest path is ${\mathcal {O}}(mn)$ in ${\mathrm {DCT}}_m$. In Sect. 4.3 we have seen that there is no algorithm with better worst-case running time for computing shortest paths. However, it might be possible to compute distances more efficiently. In fact, we established in Sect. 4.2 a way for computing distances between caterpillar trees in ${\mathcal {O}}(n \sqrt{\log n})$. This raises the question whether there is an algorithm that computes the distance between any two trees in ${\mathrm {DCT}}_m$ with better running time than ${\textsc {FindPath}}^+$.

Throughout this paper we consider ${\mathrm {DCT}}_m$ as a generalisation of ${\mathrm {RNNI}}$ by allowing internal nodes to have integer-valued time differences. We therefore introduced the parameter *m* to bound the height of a tree in the space of discrete coalescent trees in order to get a finite space. A different parameter $\rho$ has previously been introduced in Collienne and Gavryushkin (2021) for generalising ${\mathrm {RNNI}}$ to a space ${\mathrm {RNNI}}(\rho )$ of ranked trees, where rank and ${\mathrm {NNI}}$ moves have weights $\rho$ and one, respectively. Combining these two approaches of generalising ${\mathrm {RNNI}}$ gives a space of discrete coalescent trees where different moves have different weights. This tree space is relevant for practical applications, where for example some knowledge about the tree topology exists, but the uncertainty of the timing of internal nodes remains high. Investigating such a tree space could therefore be a next step for further studies.

Another tree space, of which ${\mathrm {DCT}}_m$ is a discretisation, is the *t*-space (Gavryushkin and Drummond 2016), where internal nodes are assigned real-valued times. For this space on time-trees no algorithm for computing shortest paths or distances is known yet. Our results, however, provide a way to approximate distances between time-trees. Therefore, time-trees first need to be discretised to become discrete coalescent trees between which the ${\mathrm {DCT}}_m$ distance can be computed and used as approximation of the distance between time-trees in *t*-space. For this it is important to notice that the parameter *m* is not relevant in applications, as distances between two trees in ${\mathrm {DCT}}_{m'}$ coincide with those in ${\mathrm {DCT}}_m$ if $m' < m$. Since the choice of *m*, and therefore the choice on how to discretise time-trees, drives the complexity of computing shortest paths (Sect. 4.3), finding a way to discretise time-trees to use our results on ${\mathrm {DCT}}_m$ can be subject of further research.
